# Membranous variety of rectal atresia - primary management in a neonate

**DOI:** 10.4103/0972-9941.72598

**Published:** 2010

**Authors:** Shalika Jayaswal, Hemanshi Shah, Keshav Murthy, Kailash Bhandarkar, Om Prakash Makhija

**Affiliations:** Department of Pediatric Surgery, T.N. Medical College and B.Y.L. Nair Charitable Hospital, Mumbai, India

**Keywords:** Membranous rectal atresia, primary management

## Abstract

Rectal atresia is a rare form of anorectal malformation, with reported incidence of 1 to 2% and membranous variety of rectal atresia is even rarer. Most reported cases have been dealt with a staged procedure which includes sigmoid colostomy. We diagnosed and classified the variety of rectal atresia by performing an X-Ray (invertogram along with the red rubber catheter *in situ*.). In lesser developed geographies where MRI is not readily available or not affordable, this simple test could be used to confirm the variety of rectal atresia. However, the usual fallacies of invertogram should be considered. Here we report a neonate with membranous variety of rectal atresia managed by transanal endoscopic fulguration using bugbee passed through the cystourethroscope, without a covering sigmoid stoma.

## INTRODUCTION

Rectal atresia is a rare form of anorectal malformation with an incidence of 1 to 2%. Membranous variety of rectal atresia is still rarer. We managed a case of membranous rectal atresia, primarily by transanal membranotomy using bugbee through the cystoscope, without a covering stoma.

## CASE REPORT

**Figure 1:**
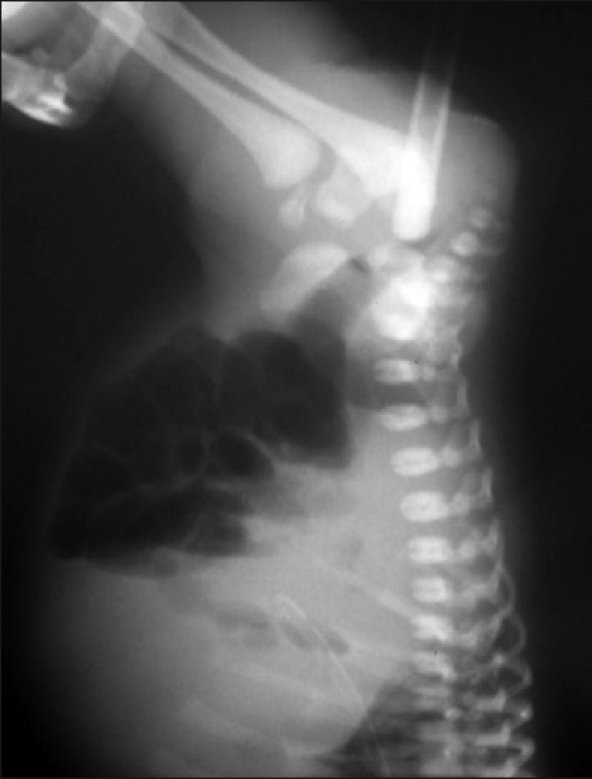
Invertogram with Red Rubber Catheter *in situ* shows catheter which is abutting the air in the bowel

A 3-day-old female child, (full-term, normal delivery) weighing 2.75 kg at birth with stable vitals, moderate abdominal distension and history of not being able to pass meconium was referred to us. Examination showed normal anal opening but red rubber catheter could not be passed beyond 3 cm of anal verge. Spine and external genitalia were normal. X-ray (invertogram with red rubber catheter passed through anal opening) showed catheter abutting the air column in the bowel) [Figure 1].

With anal retractors anal canal was opened but upper-end of membrane could not be seen.

**Figure 2:**
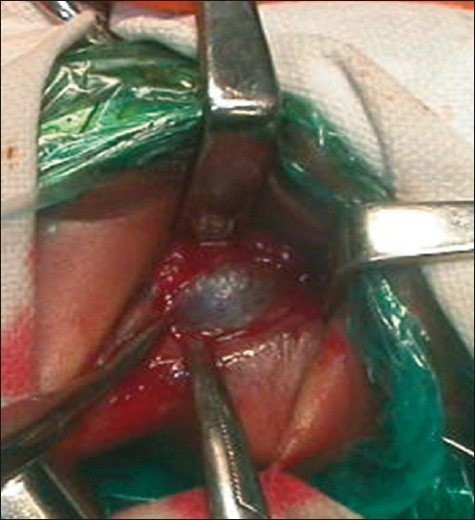
Inner mucosal membrane seen bulging

An endoscopy was done with a 7.5F cystoscope. About 3 cm from the anal verge, the anal canal ended blindly and a membrane was visualized at the blind end. We incised the outer mucosal membrane, i.e. of the distal pouch with a bugbee, but the vision was lost due to technical reasons. Then anal retractors were used, and after the incision on the mucosa of the distal pouch, the inner mucosal membrane was seen bulging significantly due to meconium within it [Figure 2].

Transanal membranotomy was done. Postoperatively, on day one, dye study was done and feeds were started. Patient was discharged on day 4.

## DISCUSSION

Rectal atresia is a rare anomaly and constitutes 1-2% of anorectal malformations with a male-female ratio of 7:3. Of the reported cases of rectal atresia, those with a membrane are even rarer. Other forms of rectal atresia include those with a fibrous cord, short-gap rectal atresia, long-gap rectal atresia and rectal stenosis.

Pathogenesis of rectal atresia is normally attributed to intravascular thrombosis secondary to intrauterine infection.

Kisra *et al*.[1] concluded that rectal atresia results from a vascular accident occurring between the 65 and 112 mm stages (13- to 14-week embryo) and that genetic factors play a minor role.

Diagnosis of rectal atresia is not difficult. A firm red rubber catheter of size 8F or 10F when passed per rectum, characteristically stops at 2-3 cm from the anal verge. X-ray (Invertogram), perineal ultrasonography and MRI have been used to verify the exact anatomy preoperatively.

In our case we diagnosed and classified the variety of rectal atresia by performing an X-Ray (invertogram along with the red rubber catheter *in situ*.).[2] In lesser developed countries where MRI is not readily available or not affordable, this simple test could be used to confirm the variety of rectal atresia. However, the usual fallacies of invertogram should be considered.

In a set of four reported cases from Japan,[3] two underwent transanal membranotomy, third underwent transverse colostomy followed by a posterior sagital ano-recto plasty (PSARP) and in the fourth case the atresia was excised using diathermy under saddle-block anesthesia.

Primary operative management is less known. Standard modality of treatment is sigmoid colostomy followed by PSARP with excision of the atretic segment and end-to-end anastomosis.

In one of the cases[4] handled by minimal invasive surgery, a colonoscope was used. This was passed through the sigmoid stoma and resection of the atretic lesion was done.

Transanal, end-to-end, rectorectal anastomosis is an upcoming technique for surgical correction of rectal atresia. In this technique, the atretic segment is intussuscepted, into the anal canal, upto the anal verge by a metal bougie, passed through the sigmoid colostomy. A midline sagittal incision exposes the rectal pouch, which is mobilised from the surrounding muscle fibers and a direct end-to-end anastomosis is performed.[5]

In most cases of rectal atresia, the anal canal and sphincter are normally formed, and therefore continence is normal after reconstruction of atresia.

Transanal endoscopic fulguration using cystourethroscope, can be used as primary management in cases of membranous rectal atresia with adjacent bowel segments.
